# Giant Abdominoscrotal Hydrocele in Adult: A Rare Entity

**DOI:** 10.7759/cureus.17562

**Published:** 2021-08-30

**Authors:** Luís Cesar Fava Spessoto, Raphael Felipe Fontes, Gabriel Beigin, Ana Clara Nagle Spessoto, Maria Fernanda Warick Facio, Fernando Nestor Facio Júnior

**Affiliations:** 1 Urology, Faculty of Medicine of São José do Rio Preto (FAMERP), São José do Rio Preto, BRA; 2 Internal Medicine, Medical School of Catanduva, Catanduva, BRA; 3 Medicine, Faceres Medical School, São José do Rio Preto, BRA

**Keywords:** hydrocele, abdominoscrotal, surgery, urology, cancer

## Abstract

Abdominoscrotal hydrocele is an uncommon condition characterized by an hourglass-shaped scrotal hydrocele with an intra-abdominal component connected by an isthmus within the inguinal canal. We report a rare case of an adult patient with giant abdominoscrotal hydrocele. Despite recent trends toward less invasive treatments, in this case, the surgical approach through an inguinal incision was the better therapeutic option with a satisfactory outcome.

## Introduction

Abdominoscrotal hydrocele (ASH) is an uncommon condition characterized by an hourglass-shaped scrotal hydrocele with an intra-abdominal component connected by an isthmus within the inguinal canal [1-3]. This is the rarest type of hydrocele [4].

Patients are generally asymptomatic, but ASH can have complications related to the compression of adjacent structures, such as hydronephrosis, hydroureter, testicular dysmorphism, testicular torsion, altered spermatogenesis, spontaneous rupture or hemorrhage and testicular malignant transformation [5,6]. As spontaneous resolution in cases of ASH is rare, early surgical intervention is recommended [4,7].

We report a rare case of an adult patient with giant ASH.

## Case presentation

**Figure 1:**
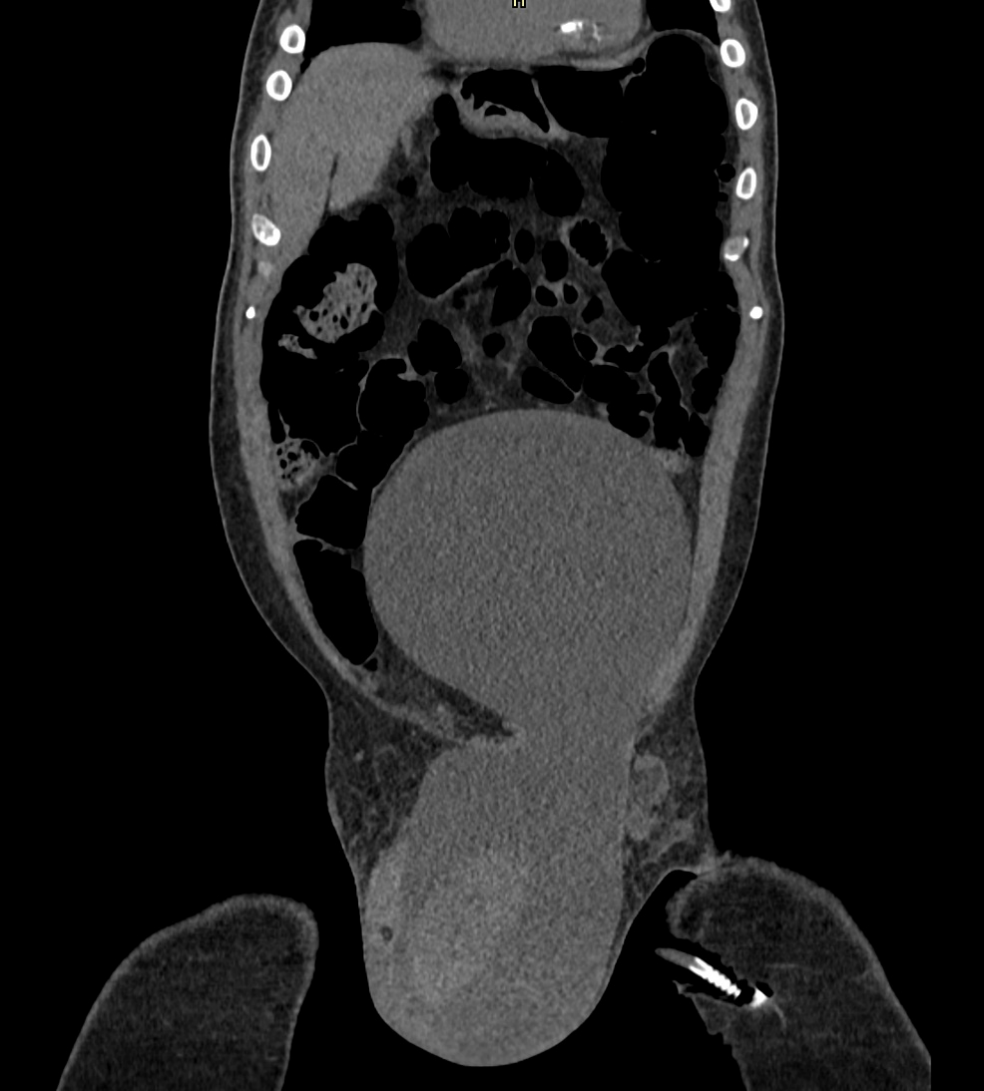
Abdominal tomogram showing voluminous cystic lesion from hypogastrium to left scrotum.

**Figure 2:**
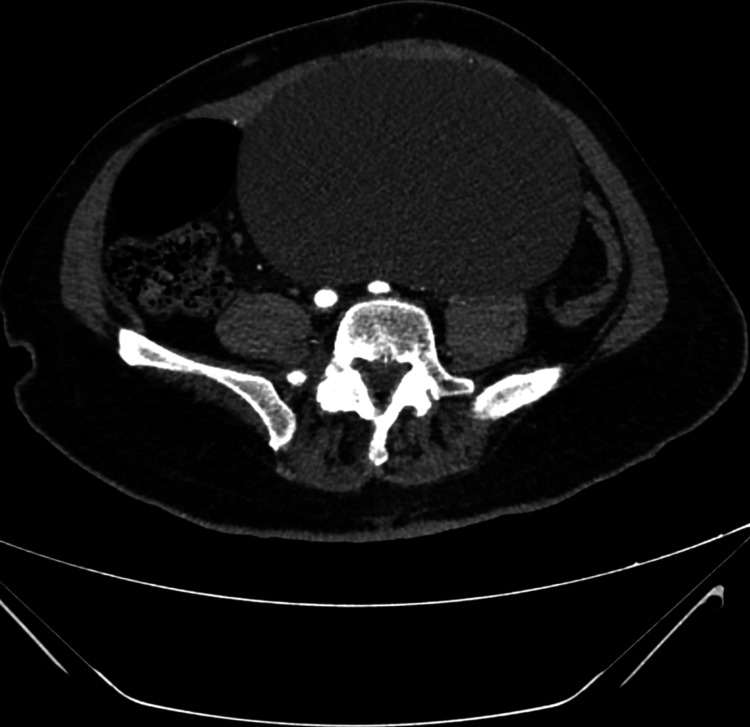
Abdominal tomogram showing voluminous cystic lesion occupying abdominal cavity with compression of adjacent structures.

**Figure 3:**
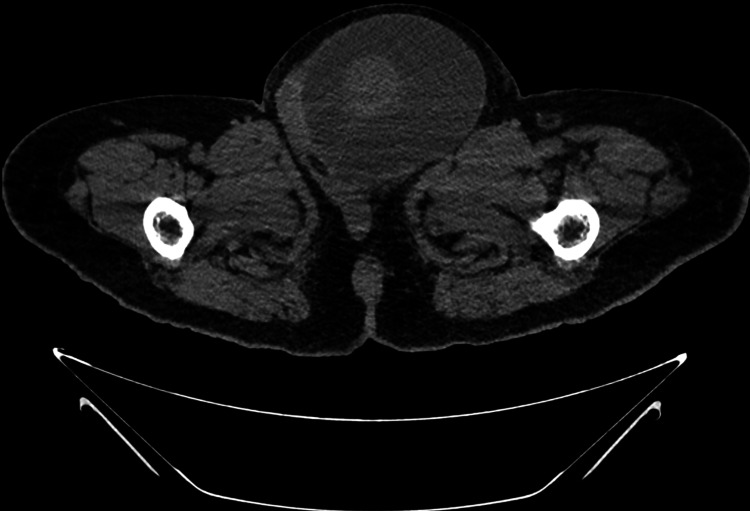
Abdominal tomogram showing left scrotum with voluminous hydrocele and increased testicle with heterogeneous enhancement.

A 59-year-old male patient with Down syndrome and obesity was admitted to the respiratory emergency room of a university hospital with dyspnea, cough and fever and was diagnosed with COVID-19. The patient had previous right-side orchiectomy due to cryptorchidism in childhood. The patient recovered well from COVID-19. During hospitalization, the patient presented with acute urinary retention associated with a palpable abdominal mass in the lower region, intestinal constipation and left-side hydrocele. The abdominoscrotal ultrasonographic exam revealed a voluminous cystic formation in the topography of the hypogastrium, with an estimated volume of 1,045 cm^3^. The results of testicular tumor markers revealed normal alpha-fetoprotein, increased human chorionic gonadotropin beta and increased lactic dehydrogenase. Computed tomography of the abdomen revealed a voluminous hourglass-shaped cystic formation involving the left testicle and extending into the abdominal cavity through the ipsilateral inguinal canal. The intra-abdominal component had an approximate volume of 2,067 cm³ and exerted compression on the bladder, rectum and sigmoid colon. The left testicle had increased dimensions and density and heterogeneous enhancement (Figures 1-3).

Left-side oncological orchiectomy was performed through inguinal access. As the hydrocele contained 3.5 L of citrine fluid, total resection was performed, causing hypogonadism. No complications occurred in the postoperative period. The anatomopathological exam was compatible with germ cell tumor (stage: TNM8, pT2). The sample was sent for histochemical analysis, which confirmed the diagnosis of seminoma.

The patient is currently in outpatient follow-up due to the diagnosis of seminoma and hypogonadism.

## Discussion

An ASH is a collection of fluid in the tunica vaginalis that extends from the scrotum into the abdominal cavity [3]. It is the least common type of hydrocele. Despite several theories, the etiology remains unknown and further studies are needed to determine the exact cause [8].

The nature of the lesion becomes apparent when a mass is felt above the inguinal ring and fluid is seen moving between the abdomen and scrotum upon compression of any of the structures [9,10].

The diagnosis of ASH is made through a clinical examination assisted by imaging methods. A positive transillumination test and cross-fluctuation between abdominal and scrotal collection are clinical benchmarks for the diagnosis. Ultrasonography is the first choice for demonstrating communication between the two components. However, computed tomography with contrast and magnetic resonance imaging can be used to demonstrate the extent of the hydrocele from the inguinal canal to the abdominal cavity [9-11].

Treatment is normally surgical, which is the recommended intervention, as a spontaneous resolution is rare and ASH can cause complications [4,7]. Different approaches have been described, such as paramedian laparotomy, inguinal access and inguinoscrotal access. The pre-peritoneal approach has been described in some cases, which facilitates the complete removal of the abdominal component [12].

## Conclusions

ASH is a rare form of hydrocele reported mainly in few case reports. ASH affects the pediatric population more than adults. In such cases, the lesion seems to be congenital. There are different hypotheses regarding its etiology as well as multiple clinical-pathological variants. A physical examination and ultrasonography are generally sufficient for the diagnosis, but further diagnostic tools may be indicated.

Despite recent trends towards less invasive treatments, surgical excision through an inguinal incision remains the standard approach to ASH. In this paper, we report a clinical case and diagnosis of a patient with ASH treated through the surgical approach.
